# Enhancing the Tensile Properties and Ductile-Brittle Transition Behavior of the EN S355 Grade Rolled Steel via Cost-Saving Processing Routes

**DOI:** 10.3390/ma17091958

**Published:** 2024-04-23

**Authors:** Vadym Zurnadzhy, Vera Stavrovskaia, Yuliia Chabak, Ivan Petryshynets, Bohdan Efremenko, Kaiming Wu, Vasily Efremenko, Michail Brykov

**Affiliations:** 1 Physics Department, Pryazovskyi State Technical University, 49000 Dnipro, Ukraine; zurnadzhy_v_i@pstu.edu (V.Z.); verochkastavrovska@gmail.com (V.S.); chabak_y_g@pstu.edu (Y.C.); efremenko_b_v@pstu.edu (B.E.); 2 Institute of Materials Research, Slovak Academy of Sciences, 04001 Kosice, Slovakia; ipetryshynets@saske.sk; 3 International Research Institute for Steel Technology, Collaborative Innovation Center for Advanced Steels, Wuhan University of Science and Technology, 430081 Wuhan, China; wukaiming@wust.edu.cn; 4 Faculty of Engineering and Physics, National University Zaporizhzhia Polytechnic, 69063 Zaporizhzhia, Ukraine; brykov@zp.edu.ua

**Keywords:** structural steel, EN S355, cost-saving, hot rolling, normalizing rolling, TMCP, accelerated cooling, microstructure, mechanical properties

## Abstract

Structural rolled steels are the primary products of modern ferrous metallurgy. Consequently, enhancing the mechanical properties of rolled steel using energy-saving processing routes without furnace heating for additional heat treatment is advisable. This study compared the effect on the mechanical properties of structural steel for different processing routes, like conventional hot rolling, normalizing rolling, thermo-mechanically controlled processing (TMCP), and TMCP with accelerating cooling (AC) to 550 °C or 460 °C. The material studied was a 20 mm-thick sheet of S355N grade (EN 10025) made of low-carbon (V+Nb+Al)-micro-alloyed steel. The research methodology included standard mechanical testing and microstructure characterization using optical microscopy, scanning and transmission electronic microscopies, energy dispersive X-ray spectrometry, and X-ray diffraction. It was found that using different processing routes could increase the mechanical properties of the steel sheets from S355N to S550QL1 grade without additional heat treatment costs. TMCP followed by AC to 550 °C ensured the best combination of strength and cold-temperature resistance due to formation of a quasi-polygonal/acicular ferrite structure with minor fractions of dispersed pearlite and martensite/austenite islands. The contribution of different structural factors to the yield tensile strength and ductile–brittle transition temperature of steel was analyzed using theoretical calculations. The calculated results complied well with the experimental data. The effectiveness of the cost-saving processing routes which may bring definite economic benefits is concluded.

## 1. Introduction

S355 grade rolled steel plate (Euronorm EN 10025) is a widely used metallurgical product [1,2,3]. S355 grade specifies that steel sheets with 16–40 mm thickness should have a yield tensile strength (YTS) of not less than 345 MPa and an ultimate tensile strength (UTS) within the 470–630 MPa interval [4]. Consequently, the total elongation (TEL) (ductility) should not be lower than 22%. This combination of mechanical properties meets the requirements in many structural applications, including civil and infrastructural construction, heavy machinery, shipbuilding, offshore, and wind farms, predetermining the widespread use of this type of flat steel products [5,6,7]. The S355 grade sheets are produced using low-carbon Mn-Si steel with micro-additions of carbide/nitride-forming elements (Nb, Ti, V, and Al). Due to lower carbon content and limited amounts of alloying elements, S355 grade steel is suitable for welded structures where the properties of the heat-affected zone are important for the integrity of the structures [8,9,10]. The EN 10025-3 [11] and EN 10025-6 [12] standards propose using a heat treatment, specifically normalization or quenching/tempering, to ensure the desired mechanical behavior in the steel of the S355–S690 grades. However, heat treatment is an energy-consumable process with significant production costs and greenhouse gas emissions.

The required steel properties must be achieved based on energy (cost)-saving solutions, which is challenging for the metallurgical industry. According to this approach, heating should be used only before rolling to form the desired final structure in the rolled steel, and no additional furnace heating should be applied for the heat treatment. The corresponding cost-saving technologies are associated with the final stages of steel sheet manufacturing, specifically with finish rolling and subsequent accelerated cooling (AC) to ensure an appropriate mechanism of the γFe → αFe phase transformation [13]. These technological processes include (a) normalizing rolling (NR) [14], (b) thermo-mechanically controlled processing (TMCP) [15], and (c) thermo-mechanically controlled processing followed by accelerated cooling (TMCP/AC) [16,17,18]. NR is a thermo-mechanical treatment with rolling completion in the lower part of the single-phase (austenite) temperature region, which inhibits the austenite grain growth and contributes to the total grain refinement. Eventually, the size of the ferrite grain after NR was close to that of furnace normalization, allowing the exclusion of this heat treatment from the technological route [19,20]. Accordingly, wide usage of NR was reported, even for manufacturing thick plates intended for offshore platform applications [21]. TMCP differs from NR by a lower completion temperature of finish rolling and a greater deformation (reduction) in the last rolling passes [22,23,24]. This effectively suppresses the austenite recrystallization, refining the ferrite grain and maintaining the strain-hardening effect (“Dislocation Engineering” [25]) with remarkably enhanced mechanical properties [26,27]. TMCP is most often used for producing high-strength steel for oil/gas pipelines [28] but is also applied while processing structural steel (S355J2 and S355G8) for more general applications [6]. Conventional hot rolling (HR), NR, and TMCP form a “ferrite + pearlite” structure with a high grade of structural banding due to a relatively slow cooling rate in still air.

A more advanced combination of properties is associated with a pearlite-free structure comprising a nonequilibrium ferrite with irregular (quasi-polygonal) [29] or acicular [30] morphologies, even lath-like bainite [31]. Bhadeshia et al. considered that the quasi-polygonal ferrite results from a diffusional phase transition having a bulk chemical composition similar to the parent austenite [32], whereas the acicular ferrite is identical to bainite regarding the transformation mechanism [33]. The acicular ferrite is characterized by high-angle boundaries, which is beneficial for the hindering of crack propagation [34]. Under a certain cooling rate, the martensite/austenite (M/A) conglomerates also appeared in the structure contributing to steel strength [35]. The pearlite-free structures mentioned earlier could be formed in low-carbon structural steel using post-rolling AC, which suppresses the austenite→pearlite transformation [36,37]. It is particularly important that this structure could be formed using heating before HR, not requiring the additional costs for furnace heating of the rolled sheets [38]. The combination of TMCP and AC led to an advanced steel strength that meets the requirements of the X80–X120 API 5L grades (Misra et al. [17,18], Ramirez et al. [39]).

Furthermore, ensuring the appropriate low-temperature impact behavior of S355 steel (especially in the transverse direction) is important since it has a wide range of applications. The absorbed impact energy (E) measured using the Charpy test controls this behavior. According to EN 10025-3, the minimum E values for longitudinal (L) and transverse (T) specimens are defined as 40 and 20 J for S355N grade, respectively, and the minimum E values for L and T specimens are defined as 47 and 27 J for S355NL grade, respectively, with the testing conducted at −20 °C in both cases. For the S355NL grade, additional testing performed at −50 °C obtained minimum E values of 27 and 16 J for L and T specimens, respectively [4]. Usually, an increased strength is associated with an increased temperature of the ductile-to-brittle transition (T_DBT_), though the “TMCP + AC” process can improve the strength and cold resistance of the rolled steel, including due to the “ferrite/pearlite” banding elimination [39]. The effectiveness of the accelerated controlled cooling after the hot rolling of high-strength structural steel was proven by Fan et al. [16], who revealed the decrease in effective grain size and increase in the dislocations density under intensification of the steel cooling. Thus, NR, TMCP, and AC in different combinations are prospective energy-saving processing routes in the final stage of steel sheet manufacturing, promoting the S355 grade steel to a much higher property level without significant additional costs. The optimal route selection should imply the comparison of different technological schemes applied to the steel sheets of the same thickness and similar chemical composition, preferably processed using the steel of the same heat. However, such comprehensive studies are rarely reported in the literature. Consequently, this study aimed to compare various technological schemes of processing the sheets made of (V+Nb+Al)-micro-alloyed structural steel, focusing on the compliance of mechanical properties with the requirements of Euronorm EN 10025. The research task was to determine the target microstructure that can be formed in the steel during its rolling and sequential cooling, in terms of gaining the optimal combination of strength and low-temperature ductile–brittle behavior.

## 2. Materials and Methods

**Figure 1:**
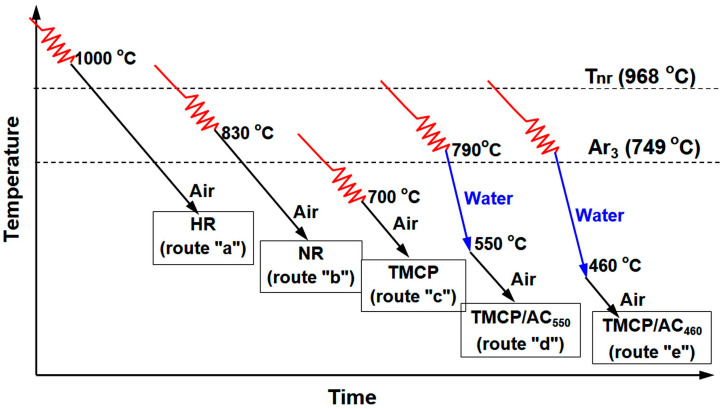
The schemes of the technological routes of S355N steel sheets processing.

The study material was S355N grade (EN 10025) rolled steel sheet. The steel contained (in wt.%) 0.12, 1.52, 0.19, 0.010, 0.010, 0.052, 0.033, 0.033, and 0.0025 of C, Mn, Si, S, P, V, Nb, Al, and Ca, respectively (Cr, Ni, and Cu were less than 0.05 wt.% each and Mo was less than 0.01 wt.%). The chemical composition and carbon equivalent of 0.39 complied with the EN 10025 requirements. The steel was smelted in a converter and cast into 220 mm thick slabs. After heating at 1180–1200 °C, the slabs were reduced (rolled) to a 20 mm thickness according to different technological routes discerned using the finish rolling temperature (FRT) and the afterward sheet cooling rate as follows (Figure 1):

- conventional HR with FRT of 980–1000 °C (which was above the non-recrystallization temperature, T_nr_) and air-cooling (route “a”);
- NR with FRT of 800–830 °C and air-cooling (route “b”);
- TMCP with FRT of 700–720 °C (in two-phase (α + γ) interval) and air-cooling (route “c”);
- TMCP/AC with FRT of 790–810 °C (in the single-phase (γFe) interval) and an accelerated cooling by water to 550 °C (route “d”) or 460 °C (route “e”).

The processing parameters were selected based on the austenite → ferrite transformation A_r3_ temperature of 748.7 °C and T_nr_ of 968.1 °C, which were calculated as follows [40]:

A_r3_ (°C) = 910 − 310C − 80Mn − 20Cu − 15Cr − 55Ni − 80Mo, (1)

(2) $$T_{\mathrm{nr}}({}^{\circ}C)=887+464C+(6445\mathrm{Nb}-644\sqrt{\mathrm{Nb}})+(732V-230\sqrt{V})+890\mathrm{Ti}+363\mathrm{Al}-357\mathrm{Si},$$

where the chemical elements were taken in wt.%.

Routes from “c” to “e” included FRT below T_nr_ to retain the strain-strengthening effect. Routes “d” and “e” had a higher FRT than route “c” to decrease the proeutectoid (polygonal) ferrite amount and increase the fraction of hard phases (acicular ferrite, bainite). Under TMCP/AC, water cooling was used to decrease the temperature of the sheet to 550 ± 20 °C (TMCP/AC_550_) or 460 ± 20 °C (TMCP/AC_460_) with a cooling intensity of 15 ± 4 and 18 ± 4 °C·s^–1^, respectively. After the completion of each processing route, the treated sheets were gathered in a stack and held for 40 h for slow cooling to 100 °C.

Mechanical properties were determined according to EN 10025 using tensile and impact tests. The longitudinal tensile specimens (5 mm in diameter and 30 mm in length gauge) were tested at room temperature with a tensile speed of 6 mm·min^–1^. The absorbed E value was measured using a Charpy pendulum-type tester on V-notched specimens of 10 mm × 10 mm × 55 mm size. The impact testing temperatures varied as 0, −20, −40, and −60 °C to define the ductile–brittle transition temperature (T_DBT_) of steel. The results were averaged using three tensile and three Charpy specimens for each regime (testing temperature). The anisotropy index (*Ai*) was calculated as follows:

(3) $$A_{i}=\frac{E_{L}}{E_{T}},$$

where, *E_L_* and *E_T_* represent the mean absorbed energy values in the longitudinal and transverse directions, respectively.

Optical microscopy (OM, Axiovert 40 MAT, Carl Zeiss, Jena, Germany) and scanning electronic microscopy (SEM, JSM-7000F, JEOL, Tokyo, Japan) were utilized for the microstructure characterization of the mirror-polished specimens etched in the 4 vol.% Nital reagent. The ferrite grain size was measured using the intercept method according to ASTM E112. The effective grain size was measured for the acicular ferrite structures. Additionally, the same SEM was used to observe the rupture surface of the impact specimen. The fine structure of the steel was characterized using transmission electron microscopy (TEM, JEM-100-C-XII, JEOL, Tokyo, Japan) equipped with energy dispersive X-ray spectroscopy (EDX, Inca-sight, Oxford Instruments, Abingdon, UK) at 100 kV acceleration voltage. For TEM, thin foils were mechanically polished to 0.1–0.15 mm thickness, followed by electropolishing in 6-vol% perchloric acid solution using a fluid-jet polishing machine. X-ray diffraction (XRD) was performed using the diffractometer (X’Pert PRO, PANalytical, Malvern, UK) with Cu-K_α_ radiation under 40 kV voltage, 50 mA tube current, 0.033° scan step, and 0.069°·s*^–^*^1^ scan speed. The broadening of ferrite peaks in XRD was used to measure the dislocation density under the assumption that dislocations caused the strain broadening in ferrite [41].

## 3. Results

### 3.1. Mechanical Properties Assessment

The tensile properties of the experimental steel, depending on the processing route, are presented in Table 1. For all cases, the strength indicators (YTS and UTS) met the requirements of EN 10025-3 for the S355N grade. After conventional HR (scheme HR), the steel had YTS, UTS, and TEL of 390 ± 6 MPa, 556 ± 8 MPa, and 27 ± 1%, respectively, fully complying with the requirements of the S355 grade. NR improved YTS by 55 MPa (to 445 ± 8 MPa), improved UTS by 14 MPa (to 570 ± 9 MPa), and increased TEL by two points (up to 29 ± 2%). Consequently, this combination of strength/ductility increased the grade of the steel to S420N. A more significant advancing of strength was achieved using TMCP with finish rolling in a two-phase temperature interval of 700–720 °C: YTS increased by 86 MPa (22.0%) and UTS increased by 20 MPa (3.6%) compared to HR. Despite the slight decrease in TEL to 25%, the mechanical properties of the TMCP-treated steel complied with the requirements of the high S460N grade. Notably, NR and TMCP had a greater impact on YTS rather than UTS. Thus, under the HR, NR, and TMCP routes with the rolled steel cooling in still air, the steel grade could be maximally increased to S460N.

The steel strength was further improved using processing schemes that combined TMCP and AC. Under TMCP/AC_550_, YTS increased by 35%, reaching 525 ± 9 MPa compared to the HR route. UTS increased by 15%, reaching 640 ± 10 MPa compared to HR, and it was a much higher increment compared to NR and TMCP. A decreased ductility (TEL of 22%) accompanied the increased strength. However, in general, the steel quality level increased to the S500Q grade, which refers to the steel subjected to the quenching-and-tempering heat treatment according to EN 10025-6. Moreover, higher strength properties were obtained (YTS of 544 ± 8 MPa and UTS of 660 ± 9 MPa) matching the level of the S550Q grade (the TEL value of 16.5% also complied with the S550Q grade) when AC reached 460 °C (TMCP/AC_460_).

**Figure 2:**
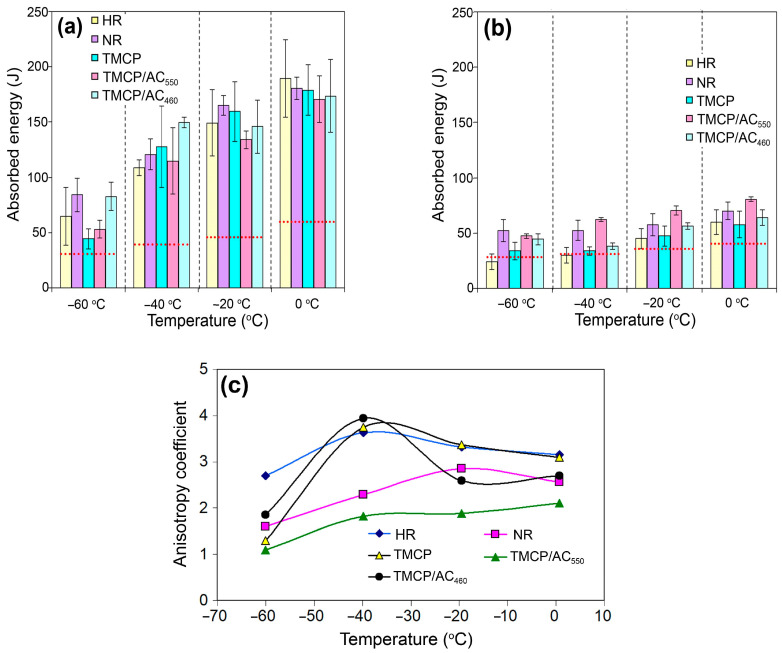
The temperature-wise evolution of the absorbed impact energy for (**a**) longitudinal and (**b**) transverse specimens. (**c**) The temperature-wise ranging of the processing routes by the anisotropy index. (Red dotted lines in (**a**,**b**) show the minimum E level corresponding to the category “QL1”).

These data on the variation in the steel quality (grade number) depending on the processing route was based on the analysis of strength properties and ductility. A more precise analysis of the data of the variation of the absorbed E depending on the testing temperatures could derive a complete picture, as graphically illustrated in Figure 2a,b. The E values gradually decreased with the decreasing testing temperature for the longitudinal and transverse specimens for each processing route, which is characteristic of the body-centered cubic (BCC) lattice [42]. The steel retained a mean absorbed energy at a high level (>100 J) in the longitudinal direction up to cooling at −40 °C, irrespective of the processing route. At −60 °C, the mean E value varied from 47 J for TMCP to 83–84 J for NR and TMCP/AC_460_. Accordingly, the absorbed impact energy at the test temperatures controlled by EN 10025-3 and EN 10025-6 (−20, −40, −50, and −60 °C) was several times higher than the minimum standard values. Under the testing at 0 °C, all processing routes ensured approximately the same E values, with some advantage for the HR specimens (Figure 2a). The NR specimens had an advantage at −20 °C. The TMCP/AC_460_ specimens had the best impact toughness at −40 and −60 °C (together with NR specimens in the latter case).

Furthermore, the transverse specimens absorbed two to three times less energy than the longitudinal specimens (Figure 2b). For different processing routes, an average E value varied from 58–81 J at 0 °C to 29–62 J at −40 °C and 24–52 J at −60 °C. At all testing temperatures, with the exception of −60 °C, the TMCP/AC_550_ specimens showed the highest mean E values. At −60 °C, TMCP/AC_550_ and NR provided the best impact behavior. In contrast, the HR and TMCP specimens absorbed the lowest energy at any testing temperature. The data showed that TMCP/AC_550_ provided the best low-temperature impact toughness in the transverse direction.

The difference in low-temperature impact behavior in longitudinal and transverse directions was assessed using *Ai*, which is the ratio of the mean absorbed energy values in the two directions (Figure 2c). Under the testing temperatures up to −40 °C, the highest *Ai* values were attributed to HR (*Ai* = 3.2–3.6), TMCP (*Ai* = 3.1–3.7), and TMCP/AC_460_ at –40 °C (*Ai* = 3.9). The lowest *Ai* value of 1.8–2.1 was attributed to the TMCP/AC_550_ specimens. Under the testing temperature of −60 °C, *Ai* reached its minimum for each processing route, and the lowest *Ai* value of 1.1 was ascribed to TMCP/AC_550_.

**Figure 3:**
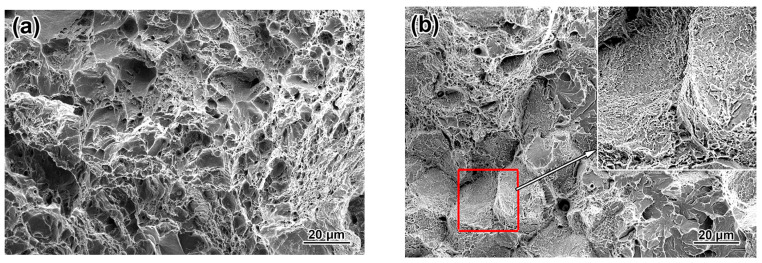
The fracture patterns of the V-notched specimens after the normalizing rolling (testing at −40 °C): (**a**) longitudinal, (**b**) transversal.

The distinction in the direction-wise impact behavior was closely related to the specific features of the fracture of the specimen. The rapture surface of the Charpy specimens tested at −40 °C (steel was subjected to NR) is illustrated in Figure 3. The fracture of the longitudinal specimen mainly consisted of dimples and tear ridges, manifesting the ductile trans-granular mechanism of the crack propagation (Figure 3a). The different sizes of the dimples indicated the micro-voids merging at higher strains, which were beneficial for the impact toughness [43]. The transverse specimen had minor dimple areas on the surface. In contrast, the inter-granular pattern combined with the quasi-cleavage areas was dominant, implying mostly brittle fracture type (Figure 3b). Notably, the inter-granular facets were not smooth and were covered with the micro-relief left after the crack branched along the grain boundaries (inset in Figure 3b). The dimples and micro-relief proved that rupture proceeded through increased absorption of energy, maintaining the E values at the acceptable level of 45–56 J.

The scatter of experimental values of absorbed energy and its mean value depending on the test temperature (the values for −50 °C were obtained using interpolation) are presented in Table 1. These data allowed for the specification of the EN 10025 grades that complied with the experimental results, specifically with respect to impact toughness. A comparison of the results with the standard norms showed that all applied processing routes provided high impact toughness (absorbed impact energy). Therefore, the steel sheet fully met the requirements of the following grades: (a) S355N and S355NL after HR, (b) S(355,420)N and S(355,420)NL after NR, (c) S(355-460)N and S(355-460)NL after TMCP, (d) S(355-460)N, S(355-460)NL, S(460,500)Q, S(460,500)QL, and S(460,500)QL1 after TMCP/AC_550_, and (e) S(355-460)N, S(355-460)NL, S(460-550)Q, S(460-550)QL, and S(460-550)QL1 after TMCP/AC_460_. Compliance of the steel with the grades marked “L” and “L1” indicated that its impact toughness met the standard over the entire controlled temperature range, including low temperatures from −40 to −60 °C, emphasizing the high quality of the steel and its compliance with the northern operational requirements.

The presented data showed that the steel strength progressively increased as the processing route changed from HR to TMCP/AC, corresponding to an increased steel grade from S355 for HR to S460 for TMCP and then to S500 and S550 for TMCP/AC_550,_ TMCP/AC_460_. Although the last two grades refer to the quenched-and-tempered steel (furnace heating), this study obtained the same high properties without the heat treatment. The ductility of steel (TEL) increased to 29% after NR, followed by a gradual decrease to 17% according to the sequence: NR → TMCP → TMCP/AC_550_ → TMCP/AC_460_. Although TEL decreased, it retained its values matching the corresponding grades that increased in the same sequence. TMCP/AC_550_ should be considered an optimal processing route for the steel studied with respect to the complex properties, like strength, ductility, low-temperature impact toughness, and anisotropy. YTS and TEL of the steel exceeded 500 MPa and 20%, respectively, for implementing TMCP/AC_550_. Additionally, the best cold resistance in the transverse direction and minimal anisotropy in impact toughness were ensured.

### 3.2. Microstructure Characterization

**Figure 4:**
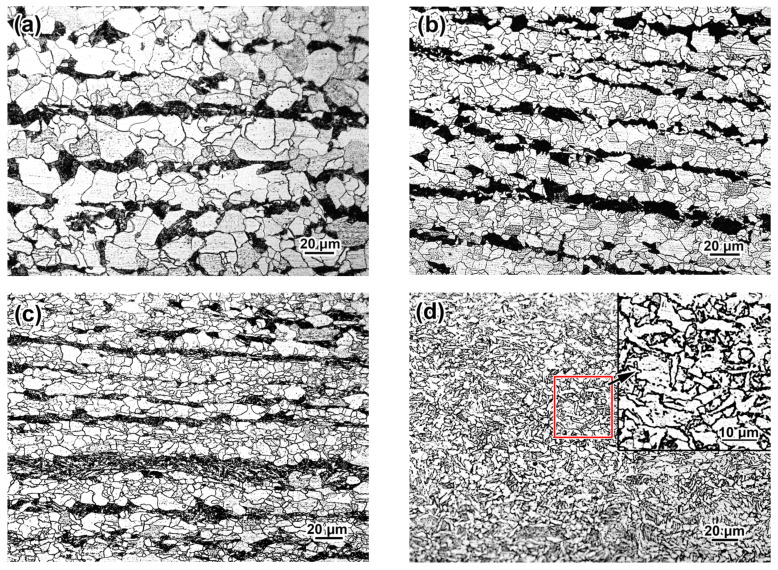

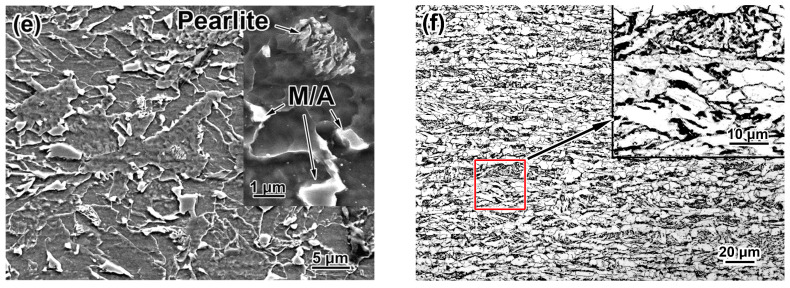
Microstructure of steel after the processing routes: (**a**) HR, (**b**) NR, (**c**) TMCP, (**d**,**e**) TMCP/AC_550_, (**f**) TMCP/AC_460_. ((**a**–**d**,**f**) are OM images, (**e**) is SEM image).

**Figure 5:**
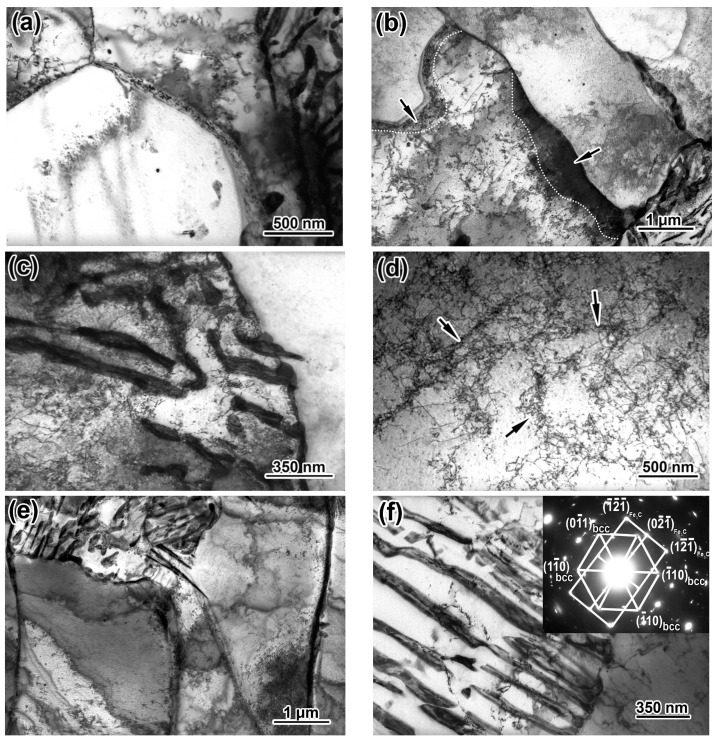

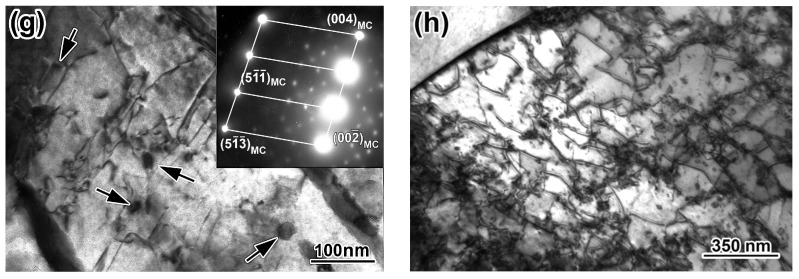
TEM images of steel after HR, NR, and TMCP treatments: (**a**,**b**,**e**) ferrite grains with a nearby pearlite colony, (**c**) pearlite colony with high dislocation density, (**d**) dislocation “walls” in ferrite grains (shown by the arrows), (**f**) cementite lamellae and selected area electron diffraction (SAED) showing the reflection on the [111] zone axis of ferrite and the reflection on the $\left[01\vec{2}\right]$ zone axis of cementite, (**g**) precipitates (Nb,V)C (shown by the arrows) and SAED showing the reflection on the [111] zone axis of carbide, (**h**) dislocation clots around the nano-precipitates inside ferrite grain. ((**a**)—HR, (**b**–**d**)—NR, (**e**–**h**)—TMCP).

The advancement in the mechanical properties of steel described earlier was due to the evolution of its microstructure on changing the processing route (Figure 4). The HR specimens had a classic “ferrite + pearlite” structure consisting of a major fraction of polygonal ferrite and 16 vol. % of pearlite (Figure 4a). Pearlite colonies were stretched along the rolling direction to form the “ferrite/pearlite” banding pattern. The ferrite grains had a near-polyhedral equiaxed shape, a non-uniform size varying from 2–3 µm to 30–36 μm, and an average diameter of 24.1 ± 1.1 µm. The grains had high-angle boundaries and a minor amount of dislocations, while the cellular substructure was not observed (Figure 5a).

NR refined the ferrite grain with the average size decreasing to 15.0 ± 0.85 µm and the grains becoming slightly elongated (Figure 4b). In addition to the fine grains, there were occasional coarse grains with up to 40 µm length and 15–17 µm width. The NR specimens retained the banding pattern, which was more pronounced compared to the HR specimens. Visually, the grains had a higher density of lattice defects with a non-uniform distribution of dislocations within the grains. Additionally, grains with a dislocation sink to the boundaries were revealed (shown using dotted lines and arrows in Figure 5b). An increased density of dislocations was also found in the ferrite layers within the pearlite colonies (Figure 5c). Many ferrite grains showed cellular substructures with the dislocation “walls” dividing the grain to the smaller sub-grains of 0.13–0.45 µm in size (0.24 ± 0.05 µm in average) (shown using arrows in Figure 5d). The heavier dislocation pattern of the NR specimens was due to finishing the deformation close to T_nr_. The dispersed precipitates of Nb and V carbides were not observed in the NR structure.

**Figure 6:**
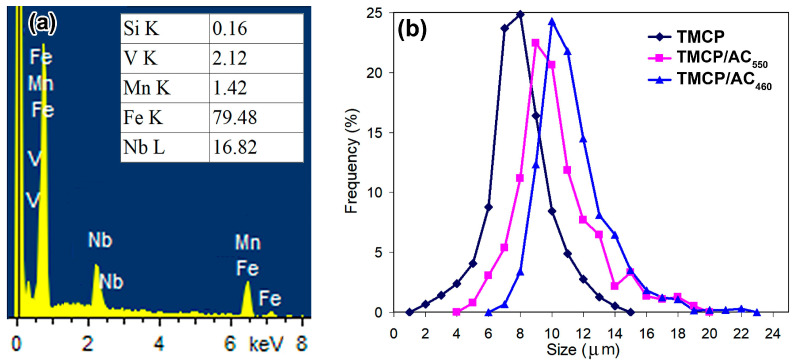
(**a**) EDS spectra and chemical composition of MC precipitate. (**b**) Size distribution of MC carbide.

TMCP further stretched and refined the ferrite grains to 12.1 ± 0.85 µm in average diameter. The grain dimension became more uniform, and the size of the coarse grains showed a maximum decrease to 25 µm length and 15–17 µm width (Figure 4c). A pronounced “ferrite/pearlite” banding characterized the TMCP specimens. The last rolling passes were performed at low temperatures of 700–720 °C under TMCP, inhibiting the dynamic and metadynamic recrystallization [44]. Additionally, this temperature range refers to the precipitation of fine V/Nb carbides [45], contributing to recrystallization suppression [46]. Subsequently, the structure acquired a heavily deformed pattern with a high dislocation density. As shown in Figure 5f, the dislocations inside the carbide plates were identified as cementite belonging to the pearlite colony, as depicted in the selected area electron diffraction (SAED) pattern in the inset to Figure 5f. As shown by the arrows in Figure 5g, the ferrite grains had fine grainy precipitates of (Nb,V)C carbide apart from the plate particles. The SAED analysis shown in the inset of Figure 5g confirmed the cubic crystal lattice (of NaCl-type) characteristic for MC carbide [47]. According to the EDX spectra in Figure 6a, the precipitates contained 16.82 wt.% of Nb, 2.12 wt.% of V, and nearly 80 wt.% of iron. The low content of strong carbide-forming elements in (Nb,V)C carbide was the artifact caused by the small size of the precipitate. Consequently, the EDX results overestimated the iron content due to the contribution of the surrounding ferric matrix [48]. The (Nb,V)C precipitates were nano-sized with an 8.1 ± 0.3 nm average value and a diameter varying in the 2–14 nm range, and 63% of the precipitate did not exceed 10 nm in size (Figure 6b). A comparison of the structures of TMCP and NR specimens showed the latter had no nano-particles and allowed to the presumption that the progressive accumulation of crystal defects due to a lower FRT under TMCP facilitated the (Nb,V)C carbide precipitation. As shown in Figure 5h, the formed precipitates interacted with dislocations using the Orowan mechanism in the grains, preferably oriented relative to the deformation direction, forming a dislocation “forest” that contributed to the steel strength [49,50].

**Figure 7:**
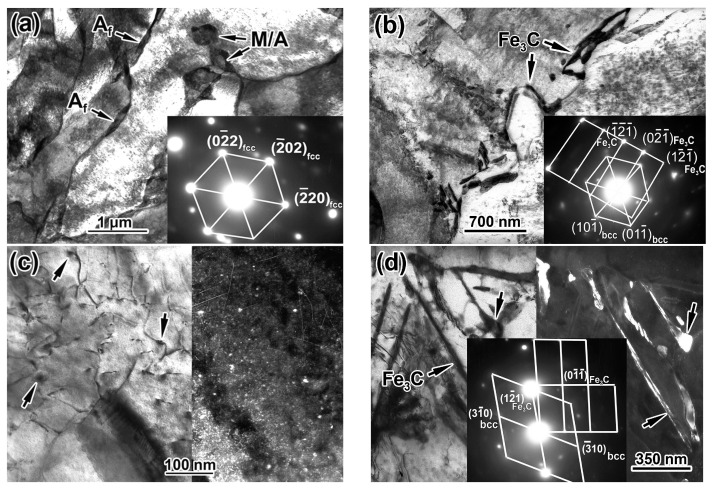
Fine microstructure (TEM images) of steel after TMCP/AC_550_ and TMCP/AC_460_ treatments: (**a**) M/A “islands” (M/A), austenite films (A_f_) in acicular ferrite grains and the SAED of M/A showing the reflection on the [111] zone axis of austenite, (**b**) cementite films and the SADEs of the reflections on the zone axes of [111] of ferrite and $\left[01\vec{2}\right]$ of cementite, (**c**) the bright-field and dark-field images of (Nb,V)C precipitates (shown by the arrows), (**d**) cementite lamellae (shown by the arrows) in bainite and the SAEDs of the reflections on the zone axes of [135] of ferrite and $\left[\bar{1}00\right]$ of cementite. ((**a**,**b**)—TMCP/AC_550_; (**c**)—TMCP/AC_460_).

The application of AC just after HR changed the structure significantly. Mostly, the TMCP/AC_550_ processing route suppressed the pearlite transformation, sharply decreasing the pearlite fraction and eliminating the “ferrite/pearlite” banding (Figure 4d). The phase transformation temperature decreased due to fast cooling. Consequently, a quasi-polygonal ferrite (irregular shape) or acicular ferrite replaced the polyhedral-shaped ferrite, with the former predominant in the structure (the area of acicular ferrite is shown in the inset in Figure 4d). The ferrite grain was additionally refined compared to TMCP, with the average size reduced to 8.9 ± 0.4 µm. The length and width of the coarsest grains did not exceed 10 and 4 µm, respectively. The colonies of fine grains of pearlite with up to 2–3 µm size were seen inside the ferrite grains. Additionally, the small grainy “islands” (0.2–2 µm) were dispersed along the grain boundaries, presumably being the martensite/austenite (M/A) conglomerates [51], as illustrated in the inset in Figure 4e. The TEM image showed the M/A “islands” as the dark-contrast bulky inclusions (denoted by single arrows in Figure 6a). SAED revealed austenite retained the in M/A constituent (inset in Figure 7a). Additionally, austenite was retained as thin films (20–95 nm) between the ferrite grains (shown by doubled arrows in Figure 7a). The SAED analysis in the inset in Figure 7b confirmed the occasional cementite films along the ferrite grains (Figure 7b). The ferrite grains had a sub-grain pattern since dislocation “walls” divided them. As shown in the left part of Figure 6c, the dislocations interacted with the nano-sized precipitates of (Nb,V)C carbide, and their dispersion is illustrated by the dark-field image in the right part of Figure 6c. The size range of the (Nb,V)C precipitates was 5–19 nm, and almost 64% of the precipitates were smaller than 10 nm (Figure 6b).

Compared to TMCP/AC_550_, the phase transformation proceeded to a lower temperature during TMCP/AC_460_, as evidenced by the appearance of approximately 25 vol. % of bainite along with irregular/acicular ferrite (Figure 4f). As shown in the TEM image in Figure 7d, thin carbide lamellae oriented from the boundaries inward of the ferrite grains characterized bainite. The dark-field observation in the cementite reflection and the corresponding SAED analysis in the inset of Figure 7d confirmed that the lamellae were cementite carbide. This observation explained the decrease in TEL to 17% compared to 22% in TMCP/AC_550_ since the borderline cementite precipitates were considered a negative factor affecting steel ductility [52]. Additionally, nano-sized (Nb,V)C precipitates were found in the structure (Figure 7c) with an approximately similar size distribution as the TMCP/AC_550_ structure (Figure 6b). The mean size of the ferrite grains after TMCP/AC_460_ was 9.9 ± 0.9 µm.

## 4. Discussion

### 4.1. The Contribution of Structural Factors to the Yield Strength of S355N Steel

The results mentioned earlier showed that using only a specific cost-saving finish processing route could significantly improve the mechanical properties of low-carbon (Nb, V, Al)-micro-alloyed steel from S355 to S550 grade. This was possible due to the inhibited grain growth and recrystallization processes, along with the decreased phase transformation temperature. The best combination of strength, ductility, and impact toughness was attributed to TMCP/AC_550_, which provided a structure mainly comprised of fine irregular-shaped ferrite grains strengthened using nano-sized carbides and M/A islands. According to Wang et al., fine and evenly dispersed M/A islands could contribute to the mechanical properties of low-carbon steel [51]. Other benefiting features of the TMCP/AC_550_ structure are (a) a predominant cementite-free state of the grain boundaries and (b) the retained austenite (as boundary-allocated films and M/A islands) that can hamper crack propagation [53] and enhance the properties due to the TRIP-effect [54,55]. These features were especially conducive to the low-temperature impact behavior of steel. Compared to TMCP/AC_550_, the precipitation of cementite carbides between the ferrite laths during bainite transformation at 460 °C (TMCP/AC_460_) decreased ductility and low-temperature toughness in the transverse direction.

The analysis of the contribution of a particular structural factor to the mechanical properties can assess its importance for steel quality. It is well known that the yield strength of low-carbon welded steels obeys the additive approach expressed as follows [28,56,57]:

(4) $$YTS=\sigma_{o}+\Delta \sigma_{SS}+\Delta \sigma_{D}+\Delta \sigma_{GB}+\Delta \sigma_{DP}+\Delta \sigma_{P}+\Delta \sigma_{M/A},$$

where *σ_o_* represents lattice friction stress (Pierls–Nabarro stress). Δ*σ_SS_* represents solid solution strengthening due to interstitial and substitutional atoms. Δ*σ_GB_*, Δ*σ_D_*, and Δ*σ_P_* represent grain boundary strengthening, dislocation strengthening, and pearlite strengthening, respectively. Δ*σ_DP_* and Δ*σ_M/A_* represent the strengthening due to dispersed precipitates and M/A islands, respectively.

The frictional stress of the α-Fe lattice (Pierls–Nabarro stress) can be roughly assessed as 2 G × 10^–4^ MPa [58]. Since the shear modulus of iron (G) is equal to 84,000 MPa, the frictional stress of the steel lattice adopted in this study was 17 MPa regardless of the steel structure.

Solid solution strengthening, which occurs due to strengthening when atoms of alloying and impurity elements are dissolved in ferrite, is calculated as follows [58]:

(5) $$\Delta \sigma_{SS}=\sum_{i=1}^{n}k_{i}c_{i},$$

where *k_i_* represents the coefficient of the *i*-element and was equal to 5440, 690, 83, 32, 30, and 30 MPa·wt%^−1^ for (C+N), P, Si, Mn, Ni, and Cr, respectively [57]. c*_i_* represents the *i*-element content in ferrite (in wt. %). Nb and V were not considered since it was presumed that they were completely bound in (Nb,V)C carbide.

Dislocation strengthening was calculated using the Taylor equation as follows [57]:

(6) $$\Delta \sigma_{D}=\alpha MGb\rho^{1/2},$$

where, *α* represents a coefficient that depends on the nature of the interaction of the dislocation during strain hardening and is equal to 0.25 [59]. *M* represents the Taylor factor that is equal to 2.73 for ferrite [57]. *G* represents the shear modulus that is equal to 81.6 GPa for ferrite [28]. *b* represents the magnitude of the Burgers vector that is equal to 0.25 nm for iron. *ρ* represents the dislocations density.

Grain boundary strengthening was estimated according to the Hall–Petch law as follows:

(7) $$\Delta \sigma_{GB}=k_{y}d^{-1/2},$$

where, *k_y_*: the Hall–Patch slope coefficient that was adopted as 0.63 MPa·m^−1/2^ for ferrite–pearlite steels [58]. *d*: average grain size.

In the case of the formation of sub-grains, sub-structural strengthening is calculated instead of grain boundary strengthening as follows [58]:

(8) $$\Delta \sigma_{S}=k_{C}l^{-m},$$

where *kc* represents the coefficient characterizing the sub-grain boundaries structure and is taken as 1.5 × 10^−4^ MPa·m. *l* represents the average sub-grain size. *m* represents the coefficient adopted as 0.5 [23].

The strengthening due to the precipitates (Δ*σ_DP_*) can be modelled using the Ashby–Orowan equation as follows [60]:

(9) $$\Delta \sigma_{DP}=\frac{6.66}{L}\ln\frac{D}{4.96\times 10^{-4}},$$

where *D* represents the mean planar intercept diameter of a precipitate. *L* represents the surface-to-surface precipitate spacing and is calculated as follows [61]:

(10) $$L=D\left[{\left(\frac{\pi }{4f}\right)}^{0.5}-1\right],$$

where *f* represents the volume fraction of the precipitates derived from TEM images.

The pearlite contribution to the strength of steel depends on its volume fraction (*P*, vol. %) as follows [58]:

(11) $$\Delta \sigma_{P}=2.4P.$$

The contribution of the M/A islands was calculated as follows:

(12) $$\Delta \sigma_{M/A}=\sigma_{M/A}f_{M/A}.$$

where *σ_M/A_* is taken as 600 MPa [62]. *f_M/_*_A_ represents the volume fraction of the M/A islands.

**Figure 8:**
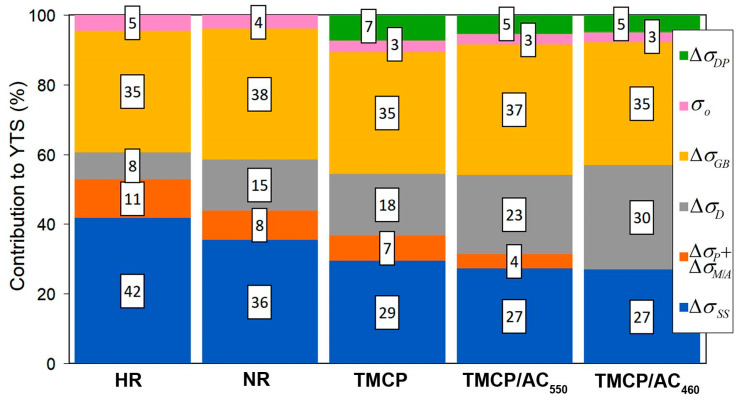
Contribution (%) of structural strengthening factors to theoretic YTS.

The theoretical yield strength for different processing routes was calculated using the values of the structural parameters presented in Table 2. The results of the calculations are depicted in Table 3 and Figure 8. Their analysis shows satisfactory compliance between theoretical results and experimental YTS values. This generally confirms the reliability of the adopted approach in understanding and assessing the factors determining the strength of structural steel. The biggest difference in results relates to the TMCP route: the calculation for TMCP gives an excess over the experimental value by 38.5 MPa (which however does not exceed 10% of the latter). A similar deviation (28.2 MPa), but with the opposite sign, refers to the HR route. These differences may be caused by an error in determining the average grain size, which depends on the location of sampling for the study. Also, in the case of TMCP, the difference may be affected by an error in the calculation of the parameters of dispersed precipitates, which were observed in the local micro-areas of the specimen. For other processing routes, the difference between the calculated data and experimental values did not exceed 15 MPa (2.8%), while the best match in the results was ascribed to TMCP/AC_550_.

As shown in Figure 8, the contribution percentage of various structural factors to the yield strength changed as the processing route changed. The solid-solution strengthening had the most significant impact of 42% in the hot-rolled state, and its contribution gradually decreased to 27% for TMCP/AC. Instead, the contribution of dislocation strengthening increased sharply from 8% for HR to 30% for TMCP/AC_460_. The latter resulted from the increased dislocation density due to the inhibited recrystallization because of the decreased FRT and the shear component of phase transition under M/A and bainite formation [63]. The “acicular ferrite + M/A + bainite” structure replaced “ferrite + pearlite”, decreasing the pearlite strengthening effect from 11% for HR to 0% for TMCP/AC_460_. The impact of grain boundaries remained stable at a high level of 35–38%, i.e., Hall–Petch strengthening was crucial for steel strength regardless of the processing mode. Although the dispersed (Nb,V)C carbides added just 28–39 MPa, they contributed indirectly through grain refinement when acting as the nuclei for the ferrite grains [64,65].

### 4.2. Variation of the Ductile–Brittle Transition Temperature Depending on the Processing Route

The evolution of a steel structure usually affects the strength and brittle fracture resistance. The latter is often assessed using T_DBT_ [64]. The change in the ductile–brittle transition temperature (Δ*T_DBT_*) of pure iron under the effect of different structural factors can be evaluated as follows [58]:

(13) $$\Delta T_{DBT}=\Delta T_{SS}+\Delta T_{D}+\Delta T_{GB}+\Delta T_{DP}+\Delta T_{P}+\Delta T_{M/A},$$

where Δ*T_SS_*, Δ*T_D_*, Δ*T_GB_*, Δ*T_DP_*, Δ*T_P_*, and Δ*T_M/A_* are the variations of T_DBT_ due to the influence of different strengthening factors such as solid solution, dislocation, dispersed precipitates, pearlite, grain boundary, and M/A islands, respectively.

It was proposed that the effect of the *i*-structural factor on Δ*T_DBT_* could be calculated by multiplying its contribution to YTS (i.e., Δ*σ_i_)* and the coefficient of embrittlement (K_i_) as follows [58]:

(14) $$\Delta T_{DBT_{i}}=\sum_{i=1}^{n}K_{i}\cdot \Delta \sigma_{i},$$

Taking the K_i_ values from [58], the Equation (13) can be presented as follows:

(15) $$\Delta T_{DBT}=0.5\cdot \Delta \sigma_{SS}+0.4\cdot \Delta \sigma_{D}+0.3\cdot \Delta \sigma_{DP}+0.9\cdot \Delta \sigma_{P}+0.5\cdot \Delta \sigma_{M/A}-0.7\cdot \Delta \sigma_{GB}.$$

**Figure 9:**
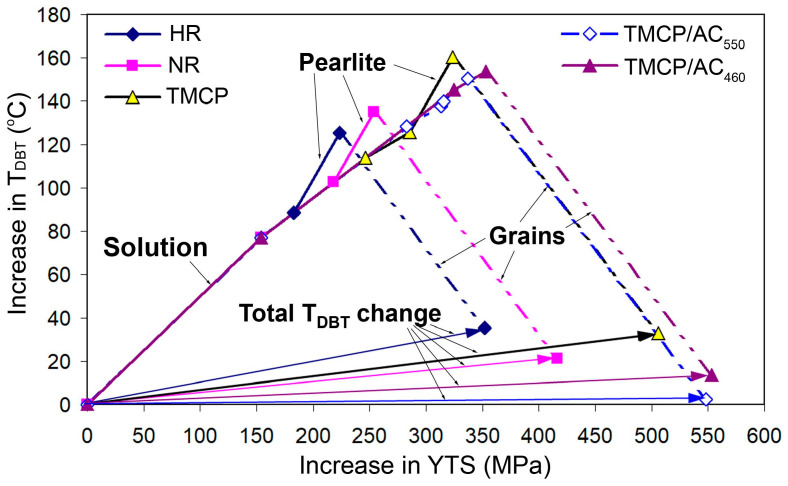
Vector diagram of the change in T_DBT_ caused by strengthening effects of different structural factors depending on the processing scheme.

The calculated results of Δ*T_DBT_* are gathered in Table 4 and shown in Figure 9 as a vector diagram. It was obvious that the majority of strengthening factors increased T_DBT_, implying steel embrittlement. However, the most deteriorating effect was associated with a solid-solution strengthening (Δ*T_P_* of 77 °C). The grain boundary strengthening was the only factor that was accompanied by a decreased T_DBT_ (Δ*T_GB_* of −90–−147 °C). As shown in the last column of Table 4 where the total Δ*T_DBT_* values are present, each of the applied processing routes should increase the cold-resistance threshold compared to pure iron due to the complex strengthening effect. In other words, the used processing routes should result in pure iron embrittlement. Consequently, the most significant increase of 35.4 and 33.0 °C in *T_DBT_* corresponds to HR and TMCP, respectively, mostly due to the presence of pearlite and increased dislocation density in the case of TMCP. Despite the presence of pearlite, T_DBT_ had a smaller increase of 21 °C after NR due to the positive effect of grain refinement. The most remarkable result implied that TMCP/AC_550_ should provide a close-to-zero change in the ductile–brittle threshold (Δ*T_DBT_* of 2.5 °C) due to further grain refinement and the replacement of pearlite by quasi-polygonal/acicular ferrite. Thus, despite a significantly increased strength compared to HR, TMCP/AC_550_ should not affect the low-temperature impact behavior of steel. The improved low-temperature behavior of the TMCP/AC_550_-treated steel in a transverse direction confirmed this conclusion. A comparison of the absorbed E for transverse specimens after HR and TMCP/AC_550_ showed that the TMCP/AC_550_ specimens had 1.5–2.0 times higher E values than HR specimens at any testing temperature (Table 1). However, TMCP/AC_460_ should somewhat worsen the low-temperature behavior of steel since T_DBT_ increased by 13.6 °C (Table 4), which is explained by an increased dislocation density due to bainite transformation. In the case of TMCP/AC_460_, grain refinement and pearlite elimination could not compensate for the dislocation-induced embrittlement. In conclusion, TMCP/AC_550_ is considered the most favorable processing route for the steel studied.

**Figure 10:**
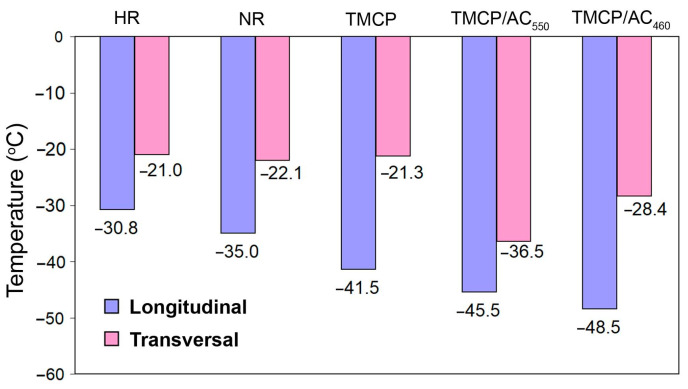
The temperature corresponding to the double reduction of the absorbed energy relative to testing at 0 °C.

There are different approaches to evaluating T_DBT_ for steel. For T_DBT_, the temperature T_50_ is often used, which corresponds to 50% ductile area on the surface of the broken specimen [66]. Additionally, T_DBT_ is estimated regarding the specified minimum value of absorbed energy [67] or T_DBT_ is taken as a temperature of the double decrease in the impact toughness relative to testing at room temperature [68]. This study followed the latter approach using the temperature dependencies of absorbed energy, as presented in Figure 3. The temperature that refers to a double decrease in the absorbed energy relative to testing at 0 °C was assessed using interpolation. The calculated values of T_DBT_ are depicted in Figure 10. T_DBT_ consistently decreased in the longitudinal direction as finish temperature decreased and post-rolling cooling intensified from –30.8 °C in the HR specimens to –48.5 °C in the TMCP/AC_460_ specimens. Although the processing route effect was less noticeable in the transverse direction, TMCP/AC_550_ stood out (T_DBT_ = −36.5 °C) and complied with the high cold resistance assessed earlier (Table 4 and Figure 9). These results proved that the combination of TMCP and AC significantly advanced the ductile-to-brittle behavior of the studied steel.

The obtained data showed that varying only the finish rolling temperature and increasing the post-rolling cooling rate could increase the yield strength of (Nb+V+Al)-micro-alloyed steel of S355 grade by about 30% from 390 to 544 MPa. The increased YTS was accompanied by the persistent or increased low-temperature impact toughness in longitudinal and transverse directions (at −20–−60 °C). Moreover, TMCP/AC decreased the rolling anisotropy, which was vital for large welded structures experiencing fatigue issues [69,70].

The results presented in this paper are based on the theoretical and experimental studies on processing routes for structural high-strength low-alloy (HSLA) steel previously fulfilled by Ishikawa et al. [71], Wang et al. [35], Misra et al. [17,18], Bhadeshia et al. [32,33], and many other researchers. The novelty of this work is that it compared different treatment modes performed on one batch of rolled steel, which neutralizes the influence of the chemical composition and makes it possible to clearly assess the influence of parameters of the technological process. This allowed us to more accurately determine the grade of rolled steel (according to EN 10025) that can be achieved depending on the processing route.

Significantly, the mechanical properties of the S355 grade steel were enhanced through a cost-saving approach that may bring definite economic benefits. This statement is supported by the rough estimation of the costs connected with steel sheet manufacturing. If we assume the hot rolling as a baseline, then NR and low-temperature TMCP (FRT of 700–720 °C) increase the production costs approximately by USD 5–7 per ton and USD 15–20 per ton, respectively, due to an increased electricity consumption. Similar cost increases can be caused by the combination of TMCP (with FRT of 790–810 °C) and accelerated water cooling. Alternatively, heat treatment with additional furnace heating (normalization) can be used to ensure the required mechanical properties. It is a much more costly operation since natural gas is consumed in large amounts (80–110 m^3^ per ton), which accounts for approximately USD 120–150 per ton. Moreover, to produce the steel sheets of S460Q-S550Q grades, the two-step “quenching-and-tempering” heat treatment is required, resulting in even higher production costs. Therefore, it is obvious that replacing heat treatment with other processing routes not associated with additional heating of the metal is economically feasible, such that it can result in savings of at least USD 100 per ton of rolled steel. Another aspect that is not mentioned yet is the possibility of further cost reduction by reducing the content of alloying elements. As can be seen from the presented data, accelerated cooling at the last stage of processing gives a significant increase in strength, which makes it possible to reduce the content of alloying and micro-alloying additives in the production of steel sheets of S355 and S410 categories. Despite the attractiveness of the cost-saving approaches, it is associated with corresponding investments. The use of the described processing routes calls for the availability of appropriate technological equipment, including a powerful rolling mill for the low-temperature deformation of steel (under NR and TMCP routes), as well as the presence of an accelerated cooling device (for TMCP/AC routes).

A limitation of the present research was studying steel sheets of only one thickness (20 mm). This does not allow evaluation of the potential capabilities of the processing routes used to improve the quality of steel-rolled products of a wider thickness range. Also, the economic benefits of using the energy-saving approaches should be assessed more precisely considering the technological peculiarities of each operation. Therefore, similar studies need to be performed for structural steel sheets of various thicknesses, including the heavy plates. This could be a possible avenue for future research on this topic.

## 5. Conclusions

This study investigated the microstructure and mechanical properties of 20 mm-thick sheets of low-carbon (V+Nb+Al)-micro-alloyed rolled steel, depending on the final manufacturing stage. Based on the obtained results, the major conclusions are drawn as follows:

1. Several processing routes without furnace heating were used at the final stage production of 20 mm-thick sheets of S355N grade steel (EN 10025), specifically HR, NR, TCMP, and AC. The variation in the processing route could increase the mechanical properties of steel from S355N to S550QL and S550QL1 grades without the additional heat treatment costs. The order of the used processing routes with the increased strength was as follows: conventional HR → NR → TMCP → TMCP/AC_550_ → TMCP/AC_460_.
2. NR improved the properties mainly through grain refinement. A further decrease in the FRT under the TMCP-included processes was accompanied by a progressive grain refinement of up to 10.5–11 numbers of ASTM E112 and an increased dislocation density that formed a sub-grained structure. The accumulation of lattice crystal defects stimulated the precipitation of nano-sized particles (2–20 nm) of (Nb,V)C carbide, which further interacted with dislocations using the Orowan mechanism.
3. The application of AC with a cooling intensity of 15–18 °C·s^−1^ after TMCP suppressed the formation of pearlite and eliminated the ferrite + pearlite structural banding. TMCP/AC_550_ formed a structure consisting of quasi-polygonal and acicular ferrite with minor fractions of dispersed pearlite and M/A islands. It ensured an optimal combination of strength (YTS of 525 MPa), ductility (TEL of 22%), and sub-zero absorbed impact energy of 115 and 62 J in the longitudinal and transversal specimens at −40 °C, respectively. The appearance of bainite under TMCP/AC_460_ led to a moderately decreased ductility (TEL of 17%) and transversal sub-zero impact toughness, which was associated with the appearance of cementite lamellae at the grain boundaries.
4. The strengthening mechanism contribution to the yield strength was defined analytically. The solid solution and grain boundary were the primary contributors to strength, irrespective of the processing route. For TMCP, the strengthening due to dispersed precipitates accounted for less than 10% of YTS. With the decreased finish rolling temperature and the involvement of water cooling, the contribution of the dislocation mechanism increased significantly, approaching 30% after TMCP/AC_460_.
5. The effects of strengthening mechanisms on Δ*T_DBT_* for steel were calculated. TMCP/AC routes minimally affected the low-temperature impact toughness of steel compared with HR, NR, and TMCP. This output complied with the experimental data showing that TMCP/AC routes ensured the lowest values of the ductile–brittle transition threshold in the longitudinal direction (−48.5 °C, TMCP/AC_460_) and in the transversal direction (−36.5 °C, TMCP/AC_550_).

## Figures and Tables

**Table 1:** Effect of the processing route on the mechanical properties of 20 mm-thick sheets of a S355N grade. L: longitudinal specimens, T: transversal specimens. The scatter of experimental values of absorbed impact energy and its mean value (in parenthesis) are given depending on the testing temperature.

Processing Rout, Corresponding EN 10025 Grades	Direction	Tensile Testing Properties			Absorbed Impact Energy (J) under Testing at				
		YTS (MPa)	UTS (MPa)	TEL (%)	0 °C	−20 °C	−40 °C	−50 °C (Interpolation)	−60 °C
Hot rolling S355N, S355NL	L	390 ± 6	556 ± 8	27 ± 2	145–222 (189 ± 38)	96–196 (150 ± 30)	62–152 (109 ± 7)	(87)	22–114 (65 ± 29)
	T	–	–	–	35–82 (60 ± 11)	30–72 (45 ± 9)	10–65 (30 ± 7)	(27)	4–52 (24 ± 7)
Normalizing rolling S(355,420)N, S(355,420)NL	L	445 ± 8	570 ± 9	29 ± 2	151–194 (180 ± 10)	80–202 (165 ± 9)	33–194 (121 ± 14)	(103)	20–120 (84 ± 15)
	T	–	–	–	66–77 (70 ± 8)	52–61 (58 ± 10)	46–56 (52 ± 9)	(52)	36–70 (52 ± 10)
TMCP S(355-460)N, S(355-460)NL	L	476 ± 5	576 ± 10	25 ± 2	152–203 (179.0 ± 23)	131–190 (160 ± 27)	46–129 (128 ± 37)	(87)	21–129 (45 ± 20)
	T	–	–	–	32–82 (58 ± 12)	20–75 (47 ± 9)	10–60 (34 ± 9)	(34)	5–61 (34 ± 8)
TMCP/AC_550_ S(355-460)N, S(355-460)NL, S(460,500)Q, S(460,500)QL, S(460,500)QL1	L	525 ± 9	640 ± 10	22 ± 2	152–200 (171 ± 21)	57–183 (134 ± 8)	71–189 (115 ± 30)	(83)	48–61 (53 ± 8)
	T	–	–	–	71–96 (81 ± 2)	67–71 (71 ± 4)	36–71 (62 ± 2)	(50)	36–59 (47 ± 2)
TMCP/AC_460_ S(355-460)N, S(355-460)NL, S(460-550)Q, S(460-550)QL S(460-550)QL1	L	554 ± 8	660 ± 9	17 ± 2	113–203 (174 ± 37)	95–191 (146 ± 24)	115–181 (150 ± 5)	(117)	41–144 (83 ± 13)
	T	–	–	–	41–90 (64 ± 7)	34–80 (56 ± 3)	7–54 (38 ± 3)	(41)	5–71 (44 ± 5)

**Table 2:** Structural parameters used for the theoretical yield strength calculation of the studied steel.

Processing Route	*ρ*, cm^–2^	*d*, μm	*D*, nm	*f*, vol.%	*P*, vol.%	*f_M/A_*, vol.%
HR	4.2 × 10^8^	24.1	–	–	16.9	–
NR	2.1 × 10^9^	15.0	–	–	15.1	–
TMCP	4.4 × 10^9^	12.1	8.1	0.56	15.9	–
TMCP/AC_550_	8.5 × 10^9^	8.9	10.1	0.51	1.0	3.5
TMCP/AC_460_	1.5 × 10^10^	9.9	11.2	0.50	–	–

**Table 3:** The contribution of structural factors to the yield strength of experimental steel (MPa). In the last column, in parentheses, the difference in YTS is shown in % to the experimental YTS.

Processing Route	*σ_o_*	Δ*σ_SS_*	Δ*σ_D_*	Δ*σ_GB_*	Δ*σ_DP_*	Δ*σ_P_*	Δ*σ_M/A_*	YTS (Calculation)	YTS (Experiment)	YTS_calc_ − YTS_exp_
HR	17	154.1	28.5	128.3	–	40.8	–	361.7	390.0 ± 6	−28.2 (7.2%)
NR	17	154.1	63.8	162.7	–	36.0	–	433.1	445.0 ± 8	−11.9 (2.7%)
TMCP	17	154.1	92.4	181.8	39.0	38.4	–	514.5	476.0 ± 5	38.5 (8.1%)
TMCP/AC_550_	17	154.1	128.4	211.2	31.0	2.4	21.0	536.0	525.0 ± 9	11.0 (2.1%)
TMCP/AC_460_	17	154.1	170.5	200.2	28.4	–	–	559.1	544.0 ± 8	−15.1 (2.8%)

**Table 4:** The contribution of structural factors to the temperature of ductile–brittle transition (°C).

Processing Route	Δ*T_SS_*	Δ*T_D_*	Δ*T_DP_*	Δ*T_P_*	Δ*T_M/A_*	Δ*T_GB_*	Δ*T_DBT_*
HR	77.1	11.4	-	36.7	-	−89.8	35.4
NR	77.1	25.5	-	32.4	-	−113.9	21.1
TMCP	77.1	32.5	11.7	34.6	-	−127.3	33.0
TMCP/AC_550_	77.1	51.3	9.3	2.2	10.5	−147.8	2.5
TMCP/AC_460_	77.1	68.2	8.5	-	-	−140.2	13.6

## Data Availability

Data are contained within the article.

## Nomenclature

AC	accelerated cooling
E	absorbed impact energy
FRT	finish rolling temperature
HR	hot rolling
K_i_	coefficient of embrittlement
L	longitudinal direction
NR	normalizing rolling
OM	optical microscopy
SAED	selected area electron diffraction
SEM	scanning electronic microscopy
T	transverse direction
T_DBT_	ductile–brittle transition temperature
TEL	total elongation
TEM	transmission electron microscopy
TMCP	thermo-mechanically controlled processing
TMCP/AC	thermo-mechanically controlled processing followed by accelerated cooling
T_nr_	non-recrystallization temperature
UTS	ultimate tensile strength
XRD	X-ray diffraction
YTS	yield tensile strength

## References

[1] Sun J., Hensel J., Klassen J., Nitschke-Pagel T., Dilger K. (2019). Solid-state phase transformation and strain hardening on the residual stresses in S355 steel weldments. J. Mater. Process. Technol..

[2] Yu B., Chen Z., Wang P., Song X. (2023). A comparative study on the mechanical behavior of S355f steel repair-welded joints. J. Constr. Steel Res..

[3] Karnaukh S.G., Markov O.E., Kukhar V.V., Shapoval A.A. (2022). Classification of steels according to their sensitivity to fracture using a synergetic model. Int. J. Adv. Manuf. Technol..

[4] (2004). Hot Rolled Products of Structural Steels—Part 1: General Technical Delivery Conditions.

[5] Kala Z., Valeš J. (2018). Imperfection sensitivity analysis of steel columns at ultimate limit state. Arch. Civ. Mech. Eng..

[6] Igwemezie V., Mehmanparast A., Kolios A. (2018). Materials selection for XL wind turbine support structures: A corrosion-fatigue perspective. Mar. Struct..

[7] Fu L., Xu G., Yan Y., Yang J., Xie J. (2018). The application and research progress of high strength and high performance steel in building structure. IOP Conf. Ser. Mater. Sci. Eng..

[8] Rozumek D., Lewandowski J., Lesiuk G., Correia J.A. (2020). The influence of heat treatment on the behavior of fatigue crack growth in welded joints made of S355 under bending loading. Int. J. Fatigue.

[9] Brykov M.N., Petryshynets I., Džupon M., Kalinin Y.A., Efremenko V.G., Makarenko N.A., Pimenov D.Y., Kováč F. (2020). Microstructure and properties of heat affected zone in high-carbon steel after welding with fast cooling in water. Materials.

[10] Yan R., Mela K., Yang F., Bamby H.E., Veljkovic M. (2023). Equivalent material properties of the heat-affected zone in welded cold-formed rectangular hollow section connections. Thin-Walled Struct..

[11] (2004). Hot Rolled Products of Structural Steels—Part 3: Technical Delivery Conditions for Normalized/Normalized Rolled Weldable Fine Grain Structural Steel.

[12] (2004). Hot Rolled Products of Structural Steels—Part 6: Technical Delivery Conditions for Flat Products of High Yield Strength Structural Steels in the Quenched and Tempered Condition.

[13] González R., García J.O., Barbés M.A., Quintana M.J., Verdeja L.F., Verdeja J.I. (2013). Structural ultrafine grained steels obtained by advanced controlled rolling. J. Iron Steel Res. Int..

[14] Gorni A.A., Da Soares M.R.S. (2015). Microstructural evolution of the normalizing plate rolling of niobium microalloyed steels. Tecnol. Metal. Mater. Min..

[15] Buchmayr B. (2017). Thermomechanical treatment of steels—A real disruptive technology since decades. Steel Res. Int..

[16] Fan Y., Wang Q., Liu H., Wang T., Wang Q., Zhang F. (2017). Effect of controlled cooling on microstructure and tensile properties of low C Nb-Ti-containing HSLA steel for construction. Metals.

[17] Zhao J., Hu W., Wang X., Kang J., Yuan G., Di H., Misra R.D.K. (2016). Effect of microstructure on the crack propagation behavior of microalloyed 560 MPa (X80) strip during ultra-fast cooling. Mater. Sci. Eng. A.

[18] Tang S., Liu Z.Y., Wang G.D., Misra R.D.K. (2013). Microstructural evolution and mechanical properties of high strength microalloyed steels: Ultra Fast Cooling (UFC) versus Accelerated Cooling (ACC). Mater. Sci. Eng. A.

[19] Laber K.B., Dyja H. (2010). The effect of the normalizing rolling of S355J2G3 steel round bars on the selected mechanical properties of finished product. Solid State Phenom..

[20] Ilievski R., Martinova Z., Krstevski B., Maddeski J. Determination of the restart temperature for normalized rolling of C-Mn steel in plate mill. Proceedings of the VIII International Congress Machines, Technologies, Materials’2011.

[21] Banasiak M., Hornik A., Szczęch S., Majta J., Kwiecień M., Cebo-Rudnicka A., Rywotycki M., Muszka K. (2021). Effect of hot-rolled heavy section bars post-deformation cooling on the microstructure refinement and mechanical properties of microalloyed steels. Metals.

[22] Sami Z., Rayane K., Alaeddine K. (2024). Effects of thermo-mechanical parameters on microstructural and mechanical properties of API X70 steel. JOM.

[23] Costa L., Melo G., Castro N., Buschinelli A. (2022). Microstructural characterization of API 5L X65 and X70 steels manufactured by TMCP process. Tecnol. Metal. Mater. Min..

[24] Roccisano A., Nafisi S., Stalheim D., Ghomashchi R. (2021). Effect of TMCP rolling schedules on the microstructure and performance of X70 steel. Mater. Charact..

[25] Huang M.X., He B.B. (2018). Alloy design by dislocation engineering. J. Mater. Sci. Technol..

[26] Hu J., Du L.X., Xie H., Gao X.H., Misra R.D.K. (2014). Microstructure and mechanical properties of TMCP heavy plate microalloyed steel. Mater. Sci. Eng. A.

[27] Singh P., Mula S., Ghosh S.A. (2023). Grain refinement, strain hardening and fracture in thermomechanically processed ultra-strong microalloyed steel. Mater. Today Commun..

[28] Guo B., Fan L., Wang Q., Fu Z., Wang Q., Zhang F. (2016). Effect of finish rolling temperature on the microstructure and tensile properties of Nb-Ti microalloyed X90 pipeline steel. Metals.

[29] Krauss G., Thompson S.W. (1995). Ferritic microstructures in continuously cooled low- and ultralow-carbon steels. ISIJ Int..

[30] Cho L., Tselikova A., Holtgrewe K., De Moor E., Schmidt R., Findley K.O. (2022). Critical assessment 42: Acicular ferrite formation and its influence on weld metal and heat-affected zone properties of steels. Mater. Sci. Technol..

[31] De-Castro D., Eres-Castellanos A., Vivas J., Caballero F.G., San-Martín D., Capdevila C. (2022). Morphological and crystallographic features of granular and lath-like bainite in a low carbon microalloyed steel. Mater. Charact..

[32] Bhadeshia H.K.D.H. (1985). Diffusional formation of ferrite in iron and its alloys. Prog. Mater. Sci..

[33] Babu S.S., Bhadeshia H.K.D.H. (1992). Stress and the acicular ferrite transformation. Mater. Sci. Eng. A.

[34] Sun L., Liu X., Xu X., Lei S., Li H., Zhai Q. (2022). Review on niobium application in microalloyed steel. J. Iron Steel Res. Int..

[35] Wang Y., Wang Q., Liu L., Xu W. (2015). Fracture mode of martensite-austenite constituents containing multiphase steel controlled by microstructural and micromechanical aspects. Mech. Adv. Mater. Struct..

[36] Efremenko V.G., Popov E.S., Kuz’min S.O., Trufanova O.I., Efremenko A.V. (2014). Introduction of three-stage thermal hardening technology for large diameter grinding balls. Metallurgist.

[37] Kong X., Lan L. (2014). Optimization of mechanical properties of low carbon bainitic steel using TMCP and accelerated cooling. Procedia Eng..

[38] Efremenko V.G., Zotov D.S., Zurnadzhy V.I., Kussa R.A., Savenko V.I., Sagirov R.I., Bocharova O.A., Efremenko A.V. (2021). Computer modelling-based selection of accelerated cooling parameters for advanced high-strength structural steel. IOP Conf. Ser. Mater. Sci. Eng..

[39] Ramirez M.F.G., Hernández J.W.C., Ladino D.H., Masoumi M., Goldenstein H. (2021). Effects of different cooling rates on the microstructure, crystallographic features, and hydrogen induced cracking of API X80 pipeline steel. J. Mater. Res. Technol..

[40] Karjalainen L.P., Maccagno T.M., Jonas J.J. (1995). Softening and flow stress behaviour of Nb microalloyed steels during hot rolling simulation. ISIJ Int..

[41] Ungár T. (2004). Microstructural parameters from X-ray diffraction peak broadening. Scr. Mater..

[42] Zhang Y.H., Ma E., Sun J., Han W.Z. (2023). A unified model for ductile-to-brittle transition in body-centered cubic metals. J. Mater. Sci. Technol..

[43] Zurnadzhy V.I., Efremenko V.G., Wu K.M., Petryshynets I., Shimizu K., Zusin A.M., Brykov M.N., Andilakhai V.A. (2020). Tailoring strength/ductility combination in 2.5 wt% Si-alloyed middle carbon steel produced by the two-step Q-P treatment with a prolonged partitioning stage. Mater. Sci. Eng. A.

[44] Ding S., Taylor T., Khan S.A., Sato Y., Yanagimoto J. (2022). Further understanding of metadynamic recrystallization through thermomechanical tests and EBSD characterization. J. Mater. Process. Technol..

[45] Zheng Y., Wang Q., Zhu L., Han B., Guo Z., Wang B., Feng J., Lu S., Shen W., Cao R. (2022). Microstructure evolution and carbide precipitation behavior of microalloyed TS800TB steel during hot rolling and coiling processes. Mater. Sci. Eng. A.

[46] Foder J., Burja J., Klančnik G. (2021). Grain size evolution and mechanical properties of Nb, V–Nb, and Ti–Nb boron type S1100QL steels. Metals.

[47] Chabak Y., Efremenko V., Zurnadzhy V., Puchý V., Petryshynets I., Efremenko B., Fedun V., Shimizu K., Bogomol I., Kulyk V. (2022). Structural and tribological studies of “(TiC + WC)/Hardened Steel” PMMC coating deposited by air pulsed plasma. Metals.

[48] Efremenko B.V., Shimizu K., Espallargas N., Efremenko V.G., Kusumoto K., Chabak Y.G., Belik A.G., Chigarev V.V., Zurnadzhy V.I. (2020). High-temperature solid particle erosion of Cr-Ni-Fe-C arc cladded coatings. Wear.

[49] Singh P.P., Ghosh S., Mula S. (2022). Strengthening behaviour and failure analysis of hot-rolled Nb+V microalloyed steel processed at various coiling temperatures. Mater. Sci. Eng. A.

[50] Chabak Y., Efremenko B., Petryshynets I., Efremenko V., Lekatou A.G., Zurnadzhy V., Bogomol I., Fedun V., Kovaľ K., Pastukhova T. (2021). Structural and tribological assessment of biomedical 316 stainless steel subjected to pulsed-plasma surface modification: Comparison of LPBF 3D printing and conventional fabrication. Materials.

[51] Wang C., Wu X., Liu J., Xu N. (2006). Transmission electron microscopy of martensite/austenite islands in pipeline steel X70. Mater. Sci. Eng. A.

[52] Zurnadzhy V., Efremenko V., Petryshynets I., Dabalà M., Franceschi M., Wu K., Kováč F., Chabak Y., Puchy V., Brykov M. (2022). Alternative approach for the intercritical annealing of (Cr, Mo, V)-alloyed TRIP-assisted steel before austempering. Metals.

[53] Koval’ A.D., Efremenko V.G., Brykov M.N., Andrushchenko M.I., Kulikovskii R.A., Efremenko A.V. (2012). Principles for developing grinding media with increased wear resistance. Part 1. Abrasive wear resistance of iron-based alloys. J. Frict. Wear.

[54] Rykavets Z.M., Bouquerel J., Vogt J.-B., Duriagina Z.A., Kulyk V.V., Tepla T.L., Bohun L.I., Kovbasyuk T.M. (2019). Investigation of the microstructure and properties of TRIP 800 steel subjected to low-cycle fatigue. Prog. Phys. Met..

[55] Hesse O., Merker J., Brykov M., Efremenko V. (2013). Zur Festigkeit niedriglegierter Stäble mit erhöhtem Kohlenstoffgehalt gegen abrasiven Verschleiß [On the strength of low-alloy steels with increased carbon content against abrasive wear]. Tribol. Schmierungstech..

[56] Hajy Akbary F., Sietsma J., Miyamoto G., Kamikawa N., Petrov R.H., Furuhara T., Santofimia M.J. (2016). Analysis of the mechanical behavior of a 0.3C-1.6Si-3.5Mn(wt%) quenching and partitioning steel. Mater. Sci. Eng. A.

[57] Kostryzhev A., Marenych O., Killmore C., Pereloma E. (2015). Strengthening mechanisms in thermomechanically processed NbTi-microalloyed steel. Metall. Mater. Trans. A.

[58] Gol’dshtejn M.I. (1986). Metal Physics of High-Strength Alloys.

[59] Huang M., Rivera-Díaz-del-Castillo P.E.J., Bouaziz O., Van der Zwaag S. (2009). Modelling strength and ductility of ultrafine grained BCC and FCC alloys using in reversible thermodynamics. Mater. Sci. Technol..

[60] Maropoulos S., Paul J.D.H., Ridley N. (1993). Microstructure–property relationships in tempered low alloy Cr–Mo–3·5Ni–V steel. Mater. Sci. Technol..

[61] Gladman T., Mcivor I.D., Dulieu D. Microalloying 75. Proceedings of the International Symposium on High Strength Low Alloy Steels.

[62] Fan L., Zhou D., Wang T., Li S., Wang Q. (2014). Tensile properties of an acicular ferrite and martensite/austenite constituent steel with varying cooling rates. Mater. Sci. Eng. A.

[63] Yakubtsov I., Zhang R., Boyd D. (2011). Particularities of the formations of bainite and martensite/austenite phase in low carbon low alloy steels during continuous cooling. Int. J. Mater. Res..

[64] Sahay S.K., Bhadeshia H.K.D.H., Honeycombe R.W.K. (1992). Carbide precipitation and the nucleation of allotriomorphic ferrite in an Fe-W-C steel. Mater. Sci. Eng. A.

[65] Zaichuk N., Shymchuk S., Tkachuk A., Feshchuk Y., Szczot J. (2021). Structure and properties of surface bandage shelves for the gas turbine engine’s blades. Advances in Design, Simulation and Manufacturing IV, Proceedings of the 4th International Conference on Design, Simulation, Manufacturing: The Innovation Exchange, DSMIE-2021, Lviv, Ukraine, 8–11 June 2021.

[66] Moura C., Vilela J.J., Rabell E.G., Martins G.P., Gonçalves Carneiro J.R. Evaluation of the ductile-to-brittle transition temperature in steel low carbon. Proceedings of the International Nuclear Atlantic Conference—INAC 2009.

[67] Chao Y.J., Ward J.D., Sands R.G. (2007). Charpy impact energy, fracture toughness and ductile–brittle transition temperature of dual-phase 590 steel. Mater. Des..

[68] Chatterjee A., Chakrabarti D., Moitra A., Mitra R., Bhaduri A.K. (2014). Effect of normalization temperatures on ductile–brittle transition temperature of a modified 9Cr–1Mo steel. Mater. Sci. Eng. A.

[69] Ostash O.P., Kulyk V.V., Poznyakov V.D., Gaivorons’kyi O.A., Vira V.V. (2019). Influence of the modes of heat treatment on the strength and cyclic crack-growth resistance of 65G steel. Mater. Sci..

[70] Mao L., Wang W., Liu Z., Sha M., Zhang D. (2022). Investigation of the fatigue crack growth behavior of S355 steel weldments of motor hangers of high-speed trains. Eng. Fail. Anal..

[71] Ishikawa N., Endo S., Kondo J. (2006). High performance UOE linepipes. JFE Tech. Rep..

